# Decline in lung function rather than baseline lung function is associated with the development of metabolic syndrome: A six-year longitudinal study

**DOI:** 10.1371/journal.pone.0174228

**Published:** 2017-03-27

**Authors:** Soo Kyoung Kim, Ji Cheol Bae, Jong-Ha Baek, Jae Hwan Jee, Kyu Yeon Hur, Moon-Kyu Lee, Jae Hyeon Kim

**Affiliations:** 1 Department of Internal Medicine, Gyeongsang National University Hospital, Gyeongsang National University School of Medicine, Jinju, Korea; 2 Institute of Health Sciences, Gyeongsang National University Hospital, Gyeongsang National University School of Medicine, Jinju, Korea; 3 Samsung Changwon Hospital, Sungkyunkwan University School of Medicine, Changwon, Korea; 4 Department of Health Promotion Center, Samsung Medical Center, Sungkyunkwan University School of Medicine, Seoul, Korea; 5 Division of Endocrinology and Metabolism, Department of Medicine, Samsung Medical Center, Sungkyunkwan University School of Medicine, Seoul, Korea; Shanghai Diabetes Institute, CHINA

## Abstract

This study was conducted to investigate whether baseline lung function or change in lung function is associated with the development of metabolic syndrome (MS) in Koreans. We analyzed clinical and laboratory data from 3,768 Koreans aged 40–60 years who underwent medical check-ups over a six-year period between 2006 and 2012. We calculated the percent change in forced vital capacity (FVC) and forced expiratory volume in 1 s (FEV_1_) over the study period. We tested for an association between baseline lung function or lung function change during the follow-up period and the development of MS. The 533 subjects (14.1%) developed MS after the six-year follow-up. The baseline FVC and FEV_1_ were not different between the subjects who developed MS after six years and the subject without MS after six years. The percent change in FVC over six years in subjects who developed MS after six years was higher than that in subjects who did not develop MS (-5.75 [-10.19 –-1.17], -3.29 [-7.69–1.09], respectively, P = 0.001). The percent change in FVC over six years was associated with MS development after adjusting for age, sex, body mass index (BMI), glucose, HDL, triglyceride, waist circumferences (WC), and systolic blood pressure. However, these association was not significant after adjusting for change of BMI and change of WC over six years (P = 0.306). The greater change in vital capacity over six years of follow-up was associated with MS development, predominantly due to obesity and abdominal obesity. The prospective study is needed to determine the relationship between lung function decline and MS.

## Introduction

There are many studies that metabolic syndrome (MS) is associated type 2 diabetes, cardiovascular disease, and other disorders, such as chronic lung disease [1–3]. Impaired lung function is also associated with glucose intolerance or diabetes, hypertension, and cardiovascular disease [4–9].

Many recent studies have shown that decreased lung function is associated with MS in Asians [10–13]. Several studies found a positive relationship between decreased lung function and MS or abdominal obesity [14, 15]. The Strong Heart Study also concluded that impaired lung function was associated with MS; however, impaired lung function at baseline did not predict MS development [16]. The authors postulated that the association between baseline lung function and MS development during the four-year follow-up could be attributed to the effects of obesity and inflammation [16].

Another recent study evaluated the association between MS and the rate of lung function decline; they found that hypertension was associated with an accelerated decline in lung function [11]. However, most studies of lung function and MS were cross-sectional analyses and very few longitudinal studies have investigated the relationship between baseline lung function or its decline and development of MS. In this study, we sought to elucidate whether baseline lung function and its decline were associated with MS development after six years using longitudinal follow-up data.

## Methods and methods

### Subjects and measurements

This retrospective longitudinal study was approved by the Ethical Committee of Samsung Medical Center at Sungkyunkwan University, in Seoul, Korea. We retrospectively assessed clinical and laboratory data from subjects who underwent routine medical check-ups at baseline and follow-up examination six years later at the Samsung Medical Health Promotion Center. Initial data were obtained from 8,888 individuals, aged 40–60 years, who participated in comprehensive health check-ups at this six-year interval (between January 2006 and December 2012). The data from the first visit served as baseline data, and 5,120 subjects were excluded based on records from their first visit for the following reasons: 1) unavailable forced expiratory volume in 1 s (FEV_1_) or forced vital capacity (FVC) level measurements (n = 3833); 2) BMI under 18 kg/m^2^ at baseline (n = 35); or 3) unavailable data regarding components of MS (n = 732); 4) Subjects with MS at baseline (N = 520). Ultimately 3,768 individuals (mean age: 49.7 years, range: 40 to 60 years) who did not have MS at their baseline visit were enrolled in the study. Of these, 533 subjects (14.1%) developed MS after the six-year follow-up period. To evaluate the change of pulmonary function in subjects with MS at baseline, we analyzed changes of lung function in the subjects with MS at baseline after the 6-year study period as separate group.

### Measurements

Metabolic syndrome was defined using the Joint Interim Statement of the International Diabetes Federation Task Force on Epidemiology and Prevention [17]. The presence of three or more of the following five components indicated the patient had MS: (1) a WC with a modified cut-off point of 90 cm for men and 80 cm for women in Asia, which is consistent with recommendations from the International Diabetes Federation [18]; (2) a systolic blood pressure (SBP) ≥130 mmHg and/or a diastolic blood pressure (DBP) ≥85 mmHg or on antihypertensive medication; (3) TG ≥150 mg/dl; (4) HDL-C <40 mg/dl in men and <50 mg/dl in women; and (5) fasting glucose ≥100 mg/dl or on anti-diabetic medication. Insulin resistance was determined using the homeostasis model assessment for insulin resistance (HOMA-IR): fasting insulin (uU/mL) * glucose (mmol/L)/22.5. The percent change in FVC or FEV_1_ was calculated using the following equation: (final FVC or FEV_1_ –baseline FVC or FEV_1_)/baseline FVC or FEV_1_ X 100. The change of BMI and WC was also calculated with same equation.

Anthropometric and other biochemical variables were measured as described previously [1].

### Statistical analysis

Data are expressed as means ± SDs or medians (25^th^– 75^th^ percentile). Metabolic risk factors, prevalence of diabetes, and other clinical characteristics were compared between subjects with and without MS after six years of follow-up. The one way ANOVA was used to compare continuous variables and the chi-square exact test was used to detect differences between groups.

Pearson’s correlation analysis was used to generate correlation coefficients between the percent change in FVC or FEV_1_ during follow-up, baseline FVC or FEV_1_, and MS components at baseline and MS components after 6 years of follow-up were calculated for all patients. Logistic regression analyses were performed to estimate odds ratios (ORs) with 95% confidence intervals (CIs) for MS development after adjusting for other variables. The ORs for MS development were calculated for each SD decrement in the percent change of FVC or FEV_1_ after adjusting for other variables. Statistical analyses were performed using PASW 17.0 software (SPSS, Inc., Chicago, IL, USA), and *P*-values < 0.05 were considered statistically significant.

## Results

Among 3,768 subjects who did not have MS at baseline, 533 (14.1%) developed MS after six years. Basal characteristics and MS components for subjects with MS at baseline, subjects with development of MS after six years, and the subjects without MS after six years are shown in Table 1. The subjects who developed MS after six years had significantly higher baseline WC, BMI, glucose, TG, and age than the subjects without MS at baseline (Table 1). The baseline FVC and FEV_1_ were not different between the subjects who developed MS after six years and the subject without MS after six years (Table 1). The subjects with MS at baseline showed the reduced FVC and FEV_1_ compared to the subject with development of MS after six years (Table 1). The cardiovascular disease was more common in the subjects with MS at baseline than subjects with development of MS after six years.

**Table 1:** Baseline characteristics of the subjects according to development of metabolic syndrome.

	The subjects with metabolic syndrome at baseline (N = 520)	The subjects with development of metabolic syndrome after six years (N = 533)	The subject without metabolic syndrome after six years (N = 3235)	P
Age (year)	51.0 ± 5.0	50.1 ± 5.0	49.6 ± 5.0	<0.001
Sex (male, %)	454 (87.3%)	412 (77.3%)	2299 (71.1%)	<0.001
BMI (kg/m2)	26.6 ± 2.3	25.4 ± 2.5	23.5 ± 2.3	<0.001
WC (cm)	92.1 ± 6.8	87.7 ± 7.9	82.6 ± 8.1	<0.001
Smoking (current smoker)	214 (41.2%)	158 (30.4%)	790 (25.2%)	<0.001
Glucose (mmol/L)	5.7 ± 1.3	5.1 ± 0.9	4.9 ± 0.7	<0.001
Body fat (%)	24.8 ± 5.6	23.9 ± 5.4	22.0 ± 5.6	<0.001
SBP (mmHg)	119.5 ± 15.1	114.2 ± 13.8	109.1 ± 13.5	<0.001
DBP (mmHg)	74.0 ± 9.9	70.6 ± 9.2	67.7 ± 9.5	<0.001
Total cholesterol (mmol/L)	4.9 ± 0.9	4.9 ± 0.8	4.9 ± 0.8	0.511
Triglyceride (mmol/L)	2.5 ± 1.2	1.8 ± 0.9	1.3 ± 0.7	<0.001
HDL cholesterol (mmol/L)	1.2 ± 0.3	1.3 ± 0.3	1.5 ± 0.3	<0.001
Hemoglobin A_1c_ (%)	5.9 ± 0.9	5.5 ± 0.7	5.3 ± 0.5	<0.001
Insulin (uIU/ml)	7.1 ± 6.3	6.5 ± 5.9	5.9 ± 5.2	<0.001
Hs-CRP (mg/L)	0.16 ± 0.26	0.12 ± 0.39	0.13 ± 0.20	<0.001
FVC (%)	90.6 ± 10.5	93.5 ± 11.2	94.1 ± 10.9	<0.001
FEV_1_ (%)	97.2 ± 12.8	100.4 ± 13.1	100.6 ± 13.2	<0.001
FEV_1_/FVC ratio (%)	79.8 ± 6.0	80.3 ± 5.6	80.2 ± 6.3	0.011
Diabetes mellitus (%)	132 (25.4%)	31 (5.8%)	111 (3.4%)	<0.001
Cardiovascular disease (%)	30 (5.8%)	13 (2.4%)	55 (1.7%)	<0.001
Cerebrovascular disease (%)	5 (1%)	15 (0.5%)	5 (0.9%)	0.182

Abbreviations: BMI, body mass index; DBP, diastolic blood pressure; FEV_1_, forced expiratory volume in 1 second; FVC, Forced vital capacity; SBP, systolic blood pressure; WC, waist circumference.

There was a significant difference in the percent change of FVC between subjects with and without MS after follow-up (-5.75 [-10.19 –-1.17], -3.29 [-7.69–1.09], respectively, P = 0.001). The subjects who developed MS showed a larger decline in FVC over six years of follow-up than the subjects who did not developed MS after six years. We also analyzed FVC change after 6 years in subjects who had MS at baseline (n = 520). The percent change in FVC after 6 years in subjects with MS at baseline (n = 520) was -5.46 (-10.31 –-0.26), which was similar to that of subjects who developed MS after six-years of follow-up (P = 0.686), but the decline of lung function was larger than that of subjects without MS during follow-up (P < 0.001).

The percent change in FEV_1_ in subjects with and without MS after six years follow-up was -11.11 (-15.63 –-6.86) and -10.66 (-14.78 –-6.67), respectively. There was no significant difference in the percent change of FEV_1_ between the two groups (P = 0.306). The FEV_1_ change after 6 years in subjects with MS at baseline was not different from that of the subjects without MS after follow-up (data not shown).

The percent change of FVC was negatively correlated with baseline age, SBP, glucose, TG, WC, hs-CRP, HOMA-IR, BMI, and percent change of BMI. Conversely, the percent change in FVC values was positively correlated with baseline HDL‐C (Table 2). The baseline FVC was also negatively related to WC, glucose, TG, and HOMA-IR at baseline (Table 2). The percent change in FEV_1_ values was negatively correlated with baseline age and BMI. However, there were no correlations between glucose, HbA_1C,_ TG, HDL-C, SBP, WC and percent change in FEV_1_ (Table 2). The baseline FEV_1_ was negatively correlated with age, WC, glucose, TG, and HOMA-IR (Table 2).

**Table 2:** Correlation between percent changes of FVC, FEV_1_, baseline FVC, FEV_1_, and metabolic risk factors at baseline.

Variable	Percent change of FVC (n = 3768)		Percent change of FEV_1_ (n = 3768)		Baseline FVC (n = 3768)		Baseline FEV_1_ (n = 3768)	
	Correlation coefficient	*P* value	Correlation coefficient	*P* value	Correlation coefficient	*P* value	Correlation coefficient	*P* value
Age (year)	-0.224	<0.001	-0.127	<0.001	-0.039	0.016	-0.039	0.018
BMI (kg/m^2^)	-0.241	<0.001	0.037	0.001	-0.012	0.467	-0.034	0.039
Body fat (%)	-0.141	0.009	0.016	0.326	-0.102	0.001	-0.027	0.104
WC (cm)	-0.233	<0.001	0.018	0.262	-0.099	<0.001	-0.147	<0.001
SBP (mmHg)	-0.059	<0.001	0.018	0.263	-0.020	0.215	-0.010	0.548
Glucose (mmol/L)	-0.063	<0.001	-0.004	0.822	-0.068	<0.001	-0.075	<0.001
Triglyceride (mmol/L)	-0.049	0.002	0.028	0.082	-0.071	<0.001	-0.087	<0.001
HDL- cholesterol (mmol/L)	0.097	<0.001	-0.026	0.106	0.071	<0.001	0.109	<0.001
HbA_1C_ (%)	-0.035	<0.001	-0.005	0.744	-0.091	<0.001	-0.083	<0.001
Insulin (uIU/ml)	-0.042	0.003	0.036	0.068	-0.090	<0.001	-0.065	<0.001
HOMA—IR	-0.069	<0.001	0.026	0.196	-0.102	<0.001	-0.082	<0.001
hs-CRP (mg/L)	-0.033	0.009	0.006	0.695	-0.051	<0.001	-0.060	<0.001
Percent change of BMI	-0.125	<0.001	-0.129	<0.001	0.000	0.977	0.010	0.523
Percent change of WC	-0.013	0.437	-0.126	<0.001	0.078	<0.001	0.114	<0.001

Abbreviations: BMI, body mass index; FEV_1_, forced expiratory volume in 1 second; FVC, Forced vital capacity; SBP, systolic blood pressure; WC, waist circumference.

The percent change in FVC was negatively correlated with BMI, WC, SBP, glucose, TG, and HOMA-IR at six-year follow-up (Table 3). The baseline FVC was also negatively correlated with WC, glucose, HOMA-IR, and hs-CRP at six-year follow-up (Table 3). The percent change in FEV_1_ was not correlated with glucose or TG. Baseline FEV_1_ was negatively correlated with WC, glucose, and TG after six years of follow-up (Table 3).

**Table 3:** Correlation between percent changes of FVC, FEV_1_, baseline FVC, FEV_1_, and metabolic risk factors after 6 years follow-up.

Variable	Percent change of FVC		Percent change of FEV_1_		Baseline FVC		Baseline FEV_1_	
	(n = 3768)		(n = 3768)		(n = 3768)		(n = 3768)	
	Correlation coefficient	*P* value	Correlation coefficient	*P* value	Correlation coefficient	*P* value	Correlation coefficient	*P* value
BMI (kg/m^2^)	-0.278	<0.001	-0.012	0.455	-0.009	0.575	-0.021	0.192
Body fat (%)	-0.205	<0.001	-0.035	0.030	-0.058	<0.001	-0.037	0.021
WC (cm)	-0.281	<0.001	-0.054	0.001	-0.060	<0.001	-0.087	<0.001
SBP (mmHg)	-0.098	<0.001	-0.012	0.454	-0.006	0.733	-0.008	0.631
Glucose (mmol/L)	-0.087	<0.001	-0.007	0.655	-0.051	0.002	-0.062	<0.001
Triglyceride (mmol/L)	-0.087	<0.001	-0.016	0.322	-0.026	0.116	-0.048	0.004
HDL- cholesterol (mmol/L)	0.140	<0.001	0.040	0.015	0.062	<0.001	0.096	<0.001
HbA_1C_ (%)	-0.083	<0.001	-0.034	0.037	-0.079	<0.001	-0.080	<0.001
Insulin (uIU/ml)	-0.055	0.001	0.014	0.378	-0.042	0.009	-0.033	0.043
HOMA—IR	-0.103	<0.001	0.020	0.322	-0.095	<0.001	-0.087	<0.001
hs-CRP (mg/L)	-0.044	0.007	-0.003	0.868	-0.072	<0.001	-0.087	<0.001

Abbreviations: BMI, body mass index; FEV_1_, forced expiratory volume in 1 second; FVC, Forced vital capacity; SBP, systolic blood pressure; WC, waist circumference.

The baseline FVC and FEV_1_ for each 1 SD increase was not associated with MS development (Table 4). The percent change of FVC was associated with the development of MS after adjusting for confounders including age, sex, BMI, glucose, HDL, TG, WC, and SBP (Table 5). After adjusting for confounders including age, sex, BMI, glucose, HDL, TG, WC, and SBP, the odds ratio (ORs) for MS development for each 1 SD increase in percent change in FVC was 0.827 (95% CI: 0.742–0.920; Table 5). However, after adjusting the percent change of WC and percent change of BMI, this relationship was not significant (Table 5).

**Table 4:** Odds ratio for incident MS based on baseline FVC and FEV_1_ in the subjects.

Variable	ORs per 1SD increase of baseline FVC (95% CI)	P-value	ORs per 1 SD increase of baseline FEV_1_ (95% CI)	P-value
Crude Model HR (95% CI)	0.958 (0.874–1.050)	0.358	0.989 (0.903–1.084)	0.816
Adjusted HR (95% CI)				
Model 1	1.052 (0.951–1.165)	0.323	1.077 (0.957–1.194)	0.160
Model 2	1.004 (0.994–1.014)	0.486	1.004 (0.995–1.012)	0.418

* by logistic regression analysis.

Model 1: adjusted for age, sex, smoking status, BMI, WC, SBP, HDL, triglyceride, glucose level at baseline.

Model 2: adjusted for age, sex, smoking status, BMI, the change of BMI, WC, the change of WC, SBP, HDL, triglyceride, glucose level at baseline.

**Table 5:** Odds ratio for incident MS based on 1SD increment of the percent change of FVC and 1SD increment of the percent change of FEV_1_ in the subjects.

Variable	ORs per 1SD increase of percent change of FVC (95% CI)	P-value	ORs per 1 SD increase of percent change of FEV_1_ (95% CI)	P-value
Crude Model HR (95% CI)	0.725 (0.660–0.796)	0.001	0.954 (0.870–1.046)	0.316
Adjusted HR (95% CI)				
Model 1	0.824 (0.742–0.916)	0.001	0.910 (0.822–1.006)	0.066
Model 2	0.827 (0.742–0.920)	0.001	0.919 (0.829–1.018)	0.107
Model 3	0.942 (0.840–1.056)	0.306	1.034 (0.924–1.158)	0.560

* by logistic regression analysis.

Model 1: adjusted for age, sex, smoking status, BMI, waist circumference, SBP, HDL, triglyceride, glucose level at baseline.

Model 2: adjusted for age, sex, smoking status, BMI, WC, SBP, HDL, triglyceride, glucose level, and FVC or FEV_1_ at baseline.

Model 3: adjusted for age, sex, smoking status, BMI, the change of BMI, WC, the change of WC, SBP, HDL, triglyceride, glucose level, and FVC or FEV_1_ at baseline.

The decrement in FEV_1_ or baseline FEV_1_ was not associated with the development of MS after six years (Tables 4 and 5).

## Discussion

This study showed that the change in FVC over six years in subjects who developed MS after six years was higher than that in subjects who did not develop MS. Previous cross-sectional analyses have reported an inverse association between lung function and MS [9, 10, 12, 13]. These studies found that impaired lung function, particularly restrictive lung impairment, was inversely associated with MS. Our previous cross-sectional study also showed that decreased vital capacity in Korean adult male subjects was associated with MS [12]. MS prevalence was significantly higher in subjects in the lowest FVC quartile compared to those in the highest FVC quartile [12].

However, longitudinal studies about the relationship between lung function decline and MS are lacking. In a longitudinal 7-year follow-up study by Engström et al., impaired lung function at baseline was found to be a risk factor for future development of insulin resistance and diabetes mellitus [8]. However, baseline FVC and FEV_1_ were not associated with MS development, which was consistent with our study (Table 4). The Strong Heart Study also observed impaired lung function in subjects with MS and diabetes; however reduced lung function at baseline did not predict MS [16]. Our study also showed that baseline FVC was not associated with MS development after six-years. We observed the change of lung function before the development of MS was associated with MS development after adjusting for age, sex, baseline BMI, baseline WC, hypertension, smoking status, glucose, and dyslipidemia. The decline in lung function is accelerated before MS development and this accelerated rate of lung function decline is maintained after MS development, indicating that deterioration of lung function parallels MS. However, this relationship between the decline of lung function and the development of MS was insignificant after adjusting the change of BMI and WC during follow-up. To our knowledge, no study to date has examined the changes in lung function before MS development. Furthermore, few studies have examined the effects of comorbidities on lung function decline. A study by Koo et al. reported that hypertension is associated with an accelerated decline in FVC [11], and the Fremantle Diabetes study showed that poor glycemic control is related to a greater decline in lung function [4]. However, these studies examined lung function decline after diagnosis of diabetes or MS and there is no adjustment for change of BMI and WC. Cuttica et al. studied the association between cardiac structural, functional change and decline of lung function [19]. They reported that with the addition of change in BMI, the association of change in lung function and cardiac output were attenuated. In our study, the decline of lung function was more rapid in the subjects who developed the MS than in the subjects who did not developed the MS, and this was related with the change of BMI and WC. Abdominal obesity mechanically affect the diaphragm and decrease the chest wall compliance, thus the change of BMI and WC was associated with more rapid decline of lung function and also related with development of MS.

Metabolic syndrome is a complex disease and the common pathophysiologic mechanisms linking MS and airway disease are suggested through the previous studies. Although the precise mechanisms have not been established, several explanations suggested. These include obesity [15], systemic inflammation [20–22], and insulin resistance [5, 14, 23]. Previous studies have reported that fasting serum insulin levels and insulin resistance index values are inversely correlated with FVC and FEV_1_ [5, 14]. Our study also showed that fasting serum insulin and insulin resistance index (HOMA-IR) values at baseline and after six years were correlated with percent change in FVC (Tables 2 and 3). Greater change in FVC was related to high insulin levels and a high insulin resistance index. Another explanation is abdominal obesity [12, 15]: Leone et al. found that the relationship between MS and decreased lung function was predominantly due to abdominal obesity [12]. Consistent with previous studies, we observed that WC after six years of follow-up was negatively correlated with percent change in FVC, and percent change in FEV_1_. Body fat after six years was also correlated with percent change in FVC. Furthermore, after adjusting the change of BMI and change of WC over six years, the change of lung function was not related with the development of MS. This means that the association between declines in FVC and MS development during follow-up was linked to obesity and abdominal obesity. The subjects with more gain of weight during follow-up had a more decline of lung function and developed the MS after six years.

There may be another common pathway involved in lung function decline and MS development: inflammation [24, 25]. Mannino et al. showed that subjects who had restrictive airway disease had elevated C-reactive protein levels [20]. They suggested that systemic inflammatory processes could result in impaired lung function. In our study, we also found that the percent changes in FVC and baseline FVC were inversely correlated with hs-CRP at baseline and hs-CRP after six years. Our study did not suggest the other mechanisms except for obesity linking MS and airway disease, thus, further prospective studies are needed to clarify the relationship between lung function change and MS, irrespective of obesity.

The strengths of our study include the relatively large sample population and the long duration of follow-up. Furthermore, ours is the first study to evaluate the relationship between lung function change and MS development. However, our study also has several limitations. First, we did not control for several potential confounding factors, including physical activity, socio-economic status, or alcohol intake. These potential confounding factors may explain our low correlation coefficients. Second, we analyzed data from subjects who voluntarily visited a health promotion center, thus they may not represent the general population. Third, although we found statistical significance for several results, the difference in lung function decline between subjects who developed MS and those who did not after six years was small. However, we also found that subjects who developed MS after six years showed similar declines in lung function compared to those who had MS from baseline. Although six years interval for the examination of the MS development may include the percent change in FVC after MS development, our data suggest that an accelerated rate of lung function decline started before MS development and continued after MS development.

In summary, a decline in vital capacity during follow-up appears to be related to MS development in Koreans, especially related with change of BMI and WC.

Lung function deterioration paralleled the development of MS and MS components. These findings imply that common pathogenic mechanisms such as central obesity or obesity contribute to both a decline in lung function and MS development. It is needed to pay attention to lung function in subjects with MS.
